# Predictive value of fibrinogen in identifying inflammatory bowel disease in active stage

**DOI:** 10.1186/s12876-021-02040-9

**Published:** 2021-12-15

**Authors:** Xiao-Fu Chen, Yuan Zhao, Yu Guo, Zhi-Ming Huang, Xie-Lin Huang

**Affiliations:** 1 Department of Gastroenterology and Hepatology, The First Affiliated Hospital of Wenzhou Medical University, Wenzhou, 325000 China; 2 Department of Gastroenterology Surgery, The First Affiliated Hospital of Wenzhou Medical University, Wenzhou, 325000 China

**Keywords:** Fibrinogen, Inflammatory bowel disease, Activity

## Abstract

**Background:**

We aimed to externally validate for the first time the diagnostic ability of fibrinogen to identify active inflammatory bowel disease (IBD).

**Methods:**

The research totally involved 788 patients with IBD, consisted of 245 ulcerative colitis (UC) and 543 Crohn’ s disease (CD). The Mayo score and Crohn disease activity index (CDAI) assessed disease activity of UC and CD respectively. The independent association between fibrinogen and disease activity of patients with UC or CD was investigated by multivariate logistic regression analyses. Area under the receiver operating characteristic curve (AUROC) assessed the performance of various biomarkers in discriminating disease states.

**Results:**

The fibrinogen levels in active patients with IBD significantly increased compared with those in remission stage (*P* < 0.001). Fibrinogen was an independent predictor to distinguish disease activity of UC (odds ratio: 2.247, 95% confidence interval: 1.428–3.537, *P* < 0.001) and CD (odds ratio: 2.124, 95% confidence interval: 1.433–3.148, *P* < 0.001). Fibrinogen was positively correlated with the Mayo score (r = 0.529, *P* < 0.001) and CDAI (r = 0.625, *P* < 0.001). Fibrinogen had a high discriminative capacity for both active UC (AUROC: 0.806, 95% confidence interval: 0.751–0.861) and CD (AUROC: 0.869, 95% confidence interval: 0.839–0.899). The optimum cut-off values of fibrinogen 3.22 was 70% sensitive and 77% specific for active UC, and 3.87 was 77% sensitive and 81% specific for active CD respectively.

**Conclusions:**

Fibrinogen is a convenient and practical biomarker to identify active IBD.

## Background

Inflammatory bowel disease (IBD), a chronic recurrent immunologic disease caused by interaction of environmental and genetic factors, become a worldwide health care issue due to its increasingly high incidence and prevalence [1–3]. Crohn’ s disease (CD) and ulcerative colitis (UC), major types of IBD, appear to result from dysregulation of the immune system [4]. Early detection of disease activity of IBD is conducive to treating the disease timely and preventing the complications effectively so as to improve quality of life and prognosis of patients [5–7]. Endoscopic biopsy remains the golden criterion for assessing and monitoring inflammatory activity of IBD in despite of many efforts to discover new biomarkers [8–10]. However, it has limited use as a result of its invasive nature and the need to collect specimens. Therefore, in order to optimally manage patients with IBD, there is a urgent need for readily obtainable and low-cost markers that enable to evaluate the disease activity of IBD.

In recent years, the lymphocyte to monocyte ratio (LMR), platelet to lymphocyte ratio (PLR), and neutrophil to lymphocyte ratio (NLR), which were acquired from the complete blood count (CBC), had been proven to be effective indicators to predict disease activity and severity of IBD [11–14]. Besides, red blood cell distribution width (RDW) had a good ability to evaluate disease activity in CD while RDW performed badly for UC [15]. Erythrocyte sedimentation rate (ESR) and C-reactive protein (CRP) were demonstrated to be significant to early diagnose IBD and accurately monitor its disease activity [9, 16, 17]. Inflammation and coagulation were interdependent processes, each activating and propagating the other, which was crucial for the progression of IBD [18, 19]. Distinct pro-inflammatory stimuli activated the clotting cascade, which in turn propagated the inflammatory state by activating signaling pathways, or recruiting more inflammatory cells to the inflamed tissue [19]. Fibrinogen, as factor I in the coagulation process, can also modify multiple aspects of inflammatory cell function by engaging leukocytes through various cellular receptors and mechanisms [20]. However, we discovered that little was known about the value of fibrinogen in identifying IBD in active stage. Therefore, in this study, we elucidated the association of fibrinogen with IBD activity and externally validated the diagnostic ability of fibrinogen to identify active IBD.

## Methods

### Patients and data

Patients with IBD included were followed up in the First Affiliated Hospital of Wenzhou Medical University during the period which began in September 2011 and ended in September 2019. The IBD diagnosis was based on a combination of clinical features, laboratory results, radiological findings, endoscopic findings, and histopathology. Exclusion criteria were as follows: (a) other immune related diseases, (b) infections, (c) carcinoma, (d) cirrhosis, (e) renal failure, (f) heart failure, (g) respiratory failure, and (h) fibrinogen data lost at admission. According to the criteria above, 788 patients with IBD (245 UC, 543 CD) were included in this retrospective research. Data were extracted from the medical database including epidemiological characteristics such as age, sex, body mass index, and duration of disease; laboratory parameters such as neutrophil, monocyte, lymphocyte, hemoglobin, RDW, platelet, prothrombin time, international normalized ratio, ESR, CRP, and fibrinogen; and endoscopic findings. Duration of disease indicated what moment of the disease the patients were in when recruited, which ranged from 0.5 to 4.0 years in patients with UC and 0.6 to 3.5 years in patients with CD. The CBC parameters were measured including NLR, LMR, and PLR.

### Disease activity

The Crohn disease activity index (CDAI) and Mayo score assessed disease activity of CD and UC respectively. Mayo score was a comprehensive scoring system which contained defecation situation, endoscopic results, rectal bleeding, and doctor’s overall evaluation [21, 22]. Patients with UC can be simply and effectively classified by the Mayo score and the score more than 2 indicated that patients with UC were in active stage. The CDAI criteria included body weight, general health condition, hematocrit, abdominal pain severity, daily blood stool count, and complications [23]. The CDAI more than 150 indicated that patients with CD were in active stage.

### Statistical analysis

Quantitative variables were expressed median [interquartile range (IQR)], compared by Mann–Whitney U test. Categorical variables were expressed absolute numbers (frequencies), compared by Chi-square test or Fisher’ s exact test. Independent association between biomarkers and disease activity in patients with UC or CD was investigated by multivariate logistic regression analyses to calculate odds ratio with a confidence interval of 95%. The Spearman’ s correlation analysis determined the relationship of biomarkers with the IBD activity. The accuracy of biomarkers in discriminating disease states was evaluated by the area under the receiver operating characteristic curve (AUROC). Calibration of fibrinogen was evaluated by Hosmer–Lemeshow goodness of fit test for significance (*P* > 0.05). DeLong test compared AUROCs of various biomarkers [24]. The sensitivity and specificity were compared at an optimum cut-off value according to the curve. Patients in UC and CD cohorts were respectively divided into two groups by the optimum cut-off values of fibrinogen. All tests were two sided. *P* < 0.05 indicated that the difference was statistically significant. STATA (version 14.0; StataCorp, State of Texas, USA) was used for statistics.

## Results

### Baseline characteristics

A total of 788 patients diagnosed with IBD were included, consisted of 245 (31.1%) with UC and 543 (68.9%) with CD. Table 1 listed characteristics of the subjects we included. Patients in CD cohort was younger than those in UC cohort on the whole. In the UC cohort, the median age was 49 years (37–60 years), and 129 (52.7%) were male, with median disease duration of 1.7 years (0.5–4.0 years). 73 (29.8%) patients were divided into UC remission group while 172 (70.2%) were categorized as UC active group. In the CD cohort, the median age was 27 years (22–33 years), and 396 (72.9%) were male, with median disease duration of 1.8 years (0.6–3.5 years). 257 (47.3%) patients were classified into CD remission group while 286 (52.7%) were categorized as CD active group.

**Table 1:** Characteristics of the inflammatory bowel disease cohort

Parameter	UC cohort (N = 245)	CD cohort (N = 543)
Age (years)	49 (37–60)	27 (22–33)
Sex: male	129 (52.7)	396 (72.9)
BMI (kg/m^2^)	19.5 (18.4–21.2)	18.9 (17.4–20.9)
Smoking: yes	39 (15.9)	84 (15.5)
Drinking: yes	22 (9.0)	44 (8.1)
Duration of disease (years)	1.7 (0.5–4.0)	1.8 (0.6–3.5)
Endoscopic inflammatory localization of disease		
Proctitis	45 (18.4)	–
Left-side colitis	107 (43.7)	–
Extensive colitis	93 (38.0)	–
Terminal ileitis	–	130 (23.9)
Colitis	–	107 (19.7)
Ileocolitis	–	306 (56.4)
Remission stage	73 (29.8)	257 (47.3)
Active stage	172 (70.2)	286 (52.7)

Values are expressed as n (%) or median (IQR). UC, ulcerative colitis; CD, Crohn’ s disease; BMI, body mass index

### Biomarkers for disease activity

The fibrinogen, CRP, ESR, PLR, and NLR levels in patients with UC in active stage scored significantly higher than those in remission stage, whereas the LMR levels were significantly lower (all *P* < 0.001) as presented in Table 2. As Table 3 showed, in patients with CD in active stage, the fibrinogen, RDW, CRP, ESR, PLR, and NLR levels were significantly higher while the LMR levels scored significantly lower compared with those in remission stage (all *P* < 0.001). Multivariate analysis demonstrated that fibrinogen, body mass index, monocyte, and CRP were independent predictors to identify UC in active stage. In addition, fibrinogen, age, sex, body mass index, duration of disease, neutrophil, lymphocyte, hemoglobin, and CRP were independent predictors to identify CD in active stage. More details about multivariate analysis results of disease activity for patients with IBD were presented in Table 4.

**Table 2:** Epidemiology and laboratory parameters of ulcerative colitis patients, stratified by disease activity

Parameter	UC remission (N = 73)	UC active (N = 172)	*P* value
Age (years)	47 (37–57)	50 (38–61)	0.331
Sex: male	32 (43.8)	97 (56.4)	0.072
BMI (kg/m^2^)	20.6 (18.8–21.7)	19.3 (18.3–20.8)	0.001
Smoking: yes	10 (13.7)	29 (16.9)	0.536
Drinking: yes	7 (9.6)	15 (8.7)	0.828
Duration of disease (years)	2.0 (0.7–4.3)	1.1 (0.3–3.8)	0.042
Neutrophil (10^9^/L)	3.3 (2.7–4.4)	4.6 (3.4–7.2)	< 0.001
Monocyte (10^9^/L)	0.4 (0.4–0.6)	0.7 (0.5–0.9)	< 0.001
Lymphocyte (10^9^/L)	1.7 (1.5–2.2)	1.8 (1.3–2.2)	0.428
Hb (g/dL)	12.8 (12.0–13.5)	11.9 (10.4–13.2)	< 0.001
RDW (%)	13.2 (12.7–13.9)	13.3 (12.7–14.3)	0.635
Platelet (10^9^/L)	235 (201–270)	281 (214–376)	< 0.001
PT (s)	13.4 (12.9–13.7)	13.7 (13.1–14.6)	< 0.001
INR	1.0 (1.0–1.1)	1.1 (1.0–1.1)	< 0.001
Fibrinogen (g/L)	2.7 (2.3–3.2)	4.0 (3.0–5.0)	< 0.001
CRP (mg/L)	3.0 (1.5–3.2)	12.8 (3.8–32.8)	< 0.001
ESR (mm/h)	6 (2–15)	21 (11–37)	< 0.001
NLR	1.8 (1.3–2.7)	2.8 (1.9–4.2)	< 0.001
PLR	130.0 (100.0–174.1)	166.4 (121.6–222.5)	< 0.001
LMR	4.2 (3.4–5.1)	2.7 (1.8–3.5)	< 0.001

Values are expressed as n (%) or median (IQR). *UC* ulcerative colitis; *BMI* body mass index; *Hb* hemoglobin; *RDW* red cell distribution width; *PT* prothrombin time; *INR* international normalized ratio; *CRP* C-reactive protein; *ESR* erythrocyte sedimentation rate; *NLR* neutrophil to lymphocyte ratio; *PLR* platelet to lymphocyte ratio; *LMR* lymphocyte to monocyte ratio

**Table 3:** Epidemiology and laboratory parameters of Crohn’ s disease patients, stratified by disease activity

Parameter	CD remission (N = 257)	CD active (N = 286)	*P* value
Age (years)	27 (22–30)	26 (22–36)	0.641
Sex: male	185 (72.0)	211 (73.8)	0.639
BMI (kg/m^2^)	19.6 (18.3–21.6)	18.2 (16.4–20.2)	< 0.001
Smoking: yes	35 (13.6)	49 (17.1)	0.258
Drinking: yes	14 (5.4)	30 (10.5)	0.032
Duration of disease (years)	2.0 (1.0–3.5)	1.1 (0.3–3.5)	< 0.001
Neutrophil (10^9^/L)	3.5 (2.7–4.4)	5.1 (3.5–7.1)	< 0.001
Monocyte (10^9^/L)	0.5 (0.4–0.6)	0.7 (0.5–0.9)	< 0.001
Lymphocyte (10^9^/L)	1.5 (1.1–1.9)	1.2 (0.9–1.7)	< 0.001
Hb (g/dL)	13.5 (12.2–14.7)	11.5 (10.1–12.6)	< 0.001
RDW (%)	13.4 (12.7–14.9)	14.8 (13.3–16.6)	< 0.001
Platelet (10^9^/L)	246 (206–294)	328 (254–414)	< 0.001
PT (seconds)	13.5 (13.1–14.0)	14.0 (13.3–14.7)	< 0.001
INR	1.1 (1.0–1.1)	1.1 (1.0–1.2)	< 0.001
Fibrinogen (g/L)	3.0 (2.4–3.6)	4.7 (3.9–5.6)	< 0.001
CRP (mg/L)	3.0 (1.6–8.6)	30.0 (15.6–60.4)	< 0.001
ESR (mm/h)	7 (2–14)	31 (17–48)	< 0.001
NLR	2.2 (1.6–3.3)	4.1 (2.8–6.2)	< 0.001
PLR	168.3 (118.8–231.9)	264.8 (192.1–370.0)	< 0.001
LMR	3.0 (2.3–4.2)	1.9 (1.4–2.6)	< 0.001

Values are expressed as n (%) or median (IQR). CD, Crohn’ s disease; *BMI* body mass index; *Hb* hemoglobin; *RDW* red cell distribution width; *PT* prothrombin time; *INR* international normalized ratio; *CRP* C-reactive protein; *ESR* erythrocyte sedimentation rate; *NLR* neutrophil to lymphocyte ratio; *PLR* platelet to lymphocyte ratio; *LMR* lymphocyte to monocyte ratio

**Table 4:** Multivariate logistic regression analysis results of disease activity for patients with inflammatory bowel disease

		95% CI		
	OR	Lower	Upper	*P* value
UC				
BMI	0.806	0.696	0.935	0.004
Monocyte	8.848	1.662	47.111	0.011
CRP	1.099	1.035	1.168	0.002
Fibrinogen	2.247	1.428	3.537	< 0.001
CD				
Age	1.059	1.026	1.092	< 0.001
Sex: male	4.975	2.254	10.981	< 0.001
BMI	0.750	0.663	0.847	< 0.001
Duration of disease	0.865	0.780	0.961	0.007
Neutrophil	1.380	1.154	1.651	< 0.001
Lymphocyte	0.593	0.353	0.996	0.048
Hb	0.484	0.392	0.597	< 0.001
CRP	1.112	1.068	1.157	< 0.001
Fibrinogen	2.124	1.433	3.148	< 0.001

ORs and *P* values were estimated using multivariate logistic regression. Age, sex, and variables were statistically significant in the tests were included in the multivariate analysis. *OR* odds ratio; *CI* confidence interval; *UC* ulcerative colitis; *BMI* body mass index; *CRP* C-reactive protein; *CD* Crohn’s disease; *Hb* hemoglobin

### Correlation of biomarkers and disease activity

Table 5 indicated correlations among biomarkers and disease activity of IBD. Fibrinogen, RDW, ESR, CRP, NLR, and PLR had significantly positive correlations with the Mayo score of UC, whereas LMR was negatively related to the Mayo score of UC (all *P* < 0.001). Besides, the correlation analysis showed significantly positive correlations of CDAI with the fibrinogen, RDW, ESR, CRP, NLR, and PLR and negative correlations with LMR (all *P* < 0.001).

**Table 5:** Spearman correlation coefficients between biomarkers and disease activity of inflammatory bowel disease

	UC (Mayo score)		CD (CDAI)	
Biomarkers	r value	*P* value	r value	*P* value
RDW	0.147	0.022	0.361	< 0.001
ESR	0.438	< 0.001	0.636	< 0.001
CRP	0.599	< 0.001	0.753	< 0.001
NLR	0.399	< 0.001	0.444	< 0.001
PLR	0.311	< 0.001	0.428	< 0.001
LMR	− 0.499	< 0.001	− 0.452	< 0.001
Fibrinogen	0.529	< 0.001	0.625	< 0.001

*P* and r values were estimated using Spearman correlation analysis. *UC* ulcerative colitis; *CD* Crohn’ s disease; *CDAI* Crohn’ s disease activity index; *RDW* red cell distribution width; *ESR* erythrocyte sedimentation rate; *CRP* C-reactive protein; *NLR* neutrophil to lymphocyte ratio; *PLR* platelet to lymphocyte ratio; *LMR* lymphocyte to monocyte ratio

### Diagnostic performance of biomarker

**Fig. 1:**
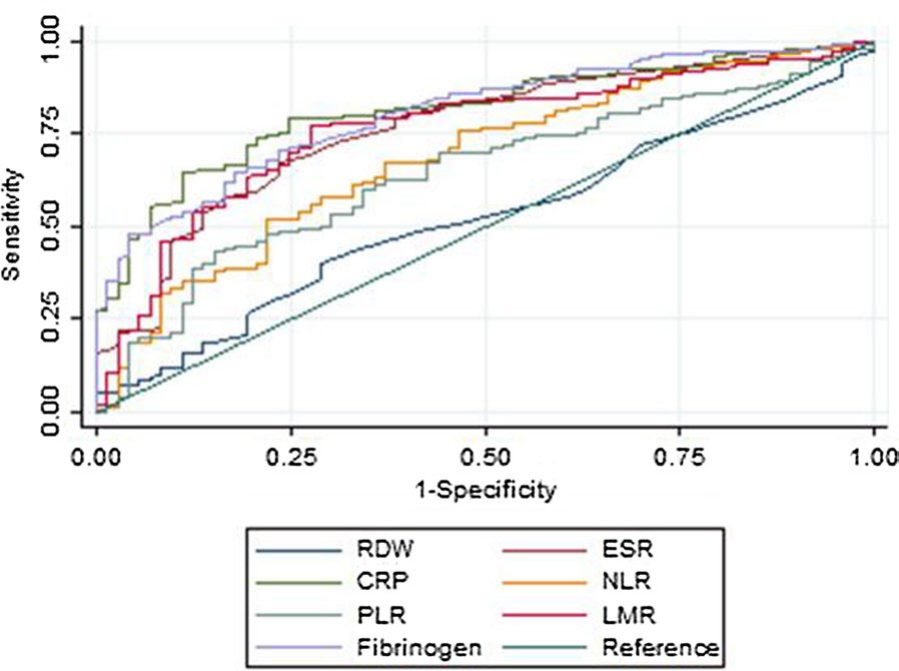
Receiver operating characteristic curves of various biomarkers in identifying active ulcerative colitis. *RDW* red cell distribution width; *ESR* erythrocyte sedimentation rate; *CRP* C-reactive protein; *NLR* neutrophil to lymphocyte ratio; *PLR* platelet to lymphocyte ratio; *LMR* lymphocyte to monocyte ratio

**Fig. 2:**
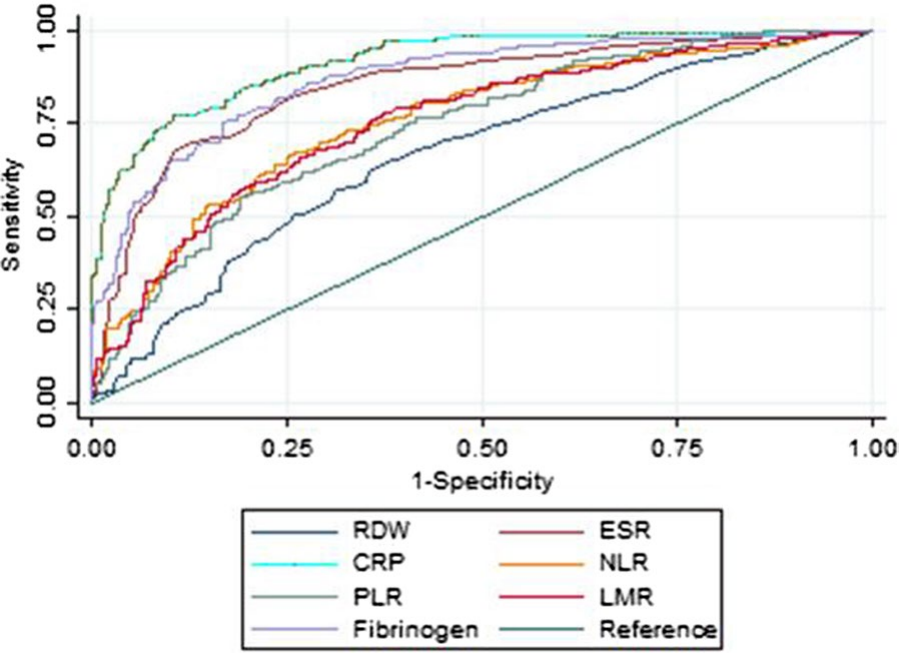
Receiver operating characteristic curves of various biomarkers in identifying active Crohn’s disease. *RDW* red cell distribution width; *ESR* erythrocyte sedimentation rate; *CRP* C-reactive protein; *NLR* neutrophil to lymphocyte ratio; *PLR* platelet to lymphocyte ratio; *LMR* lymphocyte to monocyte ratio

Figures 1 and 2 showed that fibrinogen had higher discriminative capacity for both UC and CD in active stage as compared to RDW, ESR, NLR, PLR, and LMR. CRP performed the best while RDW performed the worst. A fibrinogen cut-off value of 3.22 had a sensitivity of 70% and specificity of 77% for active UC. For active CD, a fibrinogen cut-off value of 3.87 had a sensitivity of 77% and specificity of 81%. More details about the performance of various biomarkers were presented in Tables 6 and 7. Fibrinogen had good calibration for disease activity of both UC (*P* = 0.512) and CD (*P* = 0.455).

**Table 6:** Diagnostic accuracy of various biomarkers in identifying ulcerative colitis in active stage

Biomarkers	AUROC	95% CI	*P* value	Cut-off	Sensitivity	Specificity	PV +	PV −	LR +	LR −
RDW	0.519	0.443–0.596	< 0.001	13.7	0.40	0.71	0.77	0.34	1.39	0.84
ESR	0.764	0.701–0.828	0.222	16.0	0.67	0.77	0.87	0.50	2.90	0.42
CRP	0.814	0.760–0.868	0.795	3.32	0.79	0.75	0.88	0.60	3.21	0.28
NLR	0.683	0.611–0.756	0.003	2.01	0.67	0.63	0.81	0.45	1.82	0.52
PLR	0.643	0.570–0.716	< 0.001	132	0.70	0.56	0.79	0.44	1.59	0.54
LMR	0.762	0.697–0.826	0.282	3.59	0.77	0.73	0.87	0.58	2.82	0.31
Fibrinogen	0.806	0.751–0.861	Ref	3.22	0.70	0.77	0.88	0.52	3.02	0.39

DeLong test was used to compare the AUROC between fibrinogen and other biomarkers and estimate *P* values and fibrinogen was the reference. *AUROC* area under the receiver operating characteristic curve; *CI* confidence interval; *PV* + positive predictive value; *PV* − negative predictive value; *LR* + positive likelihood ratio; *LR* − negative likelihood ratio; *RDW* red cell distribution width; *ESR* erythrocyte sedimentation rate; *CRP* C-reactive protein; *NLR* neutrophil to lymphocyte ratio; *PLR* platelet to lymphocyte ratio; *LMR* lymphocyte to monocyte ratio

**Table 7:** Diagnostic accuracy of various biomarkers in identifying Crohn’ s disease in active stage

Biomarkers	AUROC	95% CI	*P* value	Cut-off	Sensitivity	Specificity	PV +	PV −	LR +	LR −
RDW	0.661	0.615–0.707	< 0.001	14.0	0.65	0.62	0.66	0.61	1.71	0.57
ESR	0.849	0.816–0.882	0.188	21.0	0.70	0.87	0.86	0.72	5.45	0.35
CRP	0.916	0.893–0.938	< 0.001	14.8	0.77	0.89	0.89	0.78	7.32	0.26
NLR	0.758	0.717–0.798	< 0.001	3.32	0.66	0.75	0.75	0.67	2.65	0.45
PLR	0.739	0.698–0.781	< 0.001	247	0.57	0.79	0.75	0.62	2.75	0.55
LMR	0.754	0.714–0.795	< 0.001	2.67	0.79	0.61	0.69	0.73	2.04	0.34
Fibrinogen	0.869	0.839–0.899	Ref	3.87	0.77	0.81	0.82	0.76	4.12	0.28

DeLong test was used to compare the AUROC between fibrinogen and other biomarkers and estimate *P* values and fibrinogen was the reference. *AUROC* area under the receiver operating characteristic curve; *CI* confidence interval; *PV* + positive predictive value; *PV* − negative predictive value; *LR* + positive likelihood ratio; *LR* − negative likelihood ratio; *RDW* red cell distribution width; *ESR* erythrocyte sedimentation rate; *CRP* C-reactive protein; *NLR* neutrophil to lymphocyte ratio; *PLR* platelet to lymphocyte ratio; *LMR* lymphocyte to monocyte ratio

### Groups and outcomes

On the basis of the fibrinogen classification for patients with UC (Group A: < 3.22 and Group B: ≥ 3.22), the active UC patients rates among Group A and B were 47.7% (51/107), and 87.7% (121/138) respectively (*P* < 0.001). While, on the basis of the fibrinogen classification for patients with CD (Group C: < 3.87 and Group D: ≥ 3.87), the active CD patients rates among Group C and D were 24.0% (66/275), and 82.1% (220/268) respectively (*P* < 0.001). It is clear that the probability of patients with IBD in active stage significantly increased when fibrinogen was ≥ the optimal cut-off values.

## Discussion

Our study externally validated for the first time fibrinogen’ s diagnostic ability to identify patients with IBD in active stage.

It is necessary to timely determine the disease activity of IBD so that we can select optimal treatment and improve the prognosis of patients. Endoscopic biopsy is the golden criterion for assessing and monitoring inflammatory activity of IBD. As for noninvasive biomarker, fecal calprotectin prove to be currently the best biological indicator to discriminate patients with IBD and evaluate disease activity of IBD [25–28]. While, it cannot be widely used in clinical practice due to its high cost, long time requirement, and inconvenience to collect and process samples. Therefore, it is necessary and urgent to search for a simple, accessible and efficient biomarker.

Fibrinogen deposits are a near-universal feature of tissue injury including injury driven by immunological derangements [20]. IBD was an abnormal immune-mediated inflammatory disease of the intestine [2, 4]. Thus, we proposed fibrinogen, a new biomarker to identify patients with IBD in active stage. Our research found fibrinogen levels in patients with IBD in active stage significantly increased compared with those in remission stage. The correlation analysis showed significantly positive correlations of fibrinogen with the Mayo score of UC and the CDAI of CD. Through multivariate analysis to adjust for other parameters, our study further proved fibrinogen was an independent factor to distinguish disease activity of UC and CD. Fibrinogen had higher discriminative capacity for both UC (AUROC: 0.806, 0.751–0.861) and CD (AUROC: 0.869, 0.839–0.899) in active stage compared with RDW, ESR, NLR, PLR, and LMR. Besides, we found CRP performed better than fibrinogen in identifying active IBD. Fibrinogen may be a useful adjunct to or used in conjunction with CRP to quickly and timely identify active IBD. Moreover, we respectively divided patients in UC and CD cohorts into two groups by the optimum cut-off values of fibrinogen. And we found the probability of patients with IBD in active stage significantly increased when fibrinogen was ≥ the optimal cut-off values.

This biomarker has its own unique advantages. First, fibrinogen values can be easily obtained and objectively assessed with an inexpensive and noninvasive blood test. Second, fibrinogen can be used immediately without tedious calculation process. Third, this biomarker has good prediction accuracy and practicality. A fibrinogen more than 3.22 among patients with UC and 3.87 among patients with CD could be an indicator for patients being in active stage. Finally, the fibrinogen classification has some potentially clinical application value in these aspects such as screening of patients with IBD requiring treatment, evaluation of treatment effect, and prediction of IBD complications, which need further study.

However, the study had limitations. First, this research was retrospective and single-center. Besides, some factors were not taken into account such as immunosuppressant and corticosteroids use, which may have influence on the inflammatory marker levels. Finally, part of inflammatory markers were included while others were excluded. We will try to solve these problems in the following research.

## Conclusions

Among different biomarkers including fibrinogen, RDW, ESR, CRP, NLR, PLR, and LMR, fibrinogen has a high discriminative capacity for active IBD. Fibrinogen is a convenient and practical biomarker to identify patients with IBD in active stage. Further work is warranted to explore and verify other potentially clinical applications of fibrinogen.

## Data Availability

The datasets used and analysed during the current study are available from the corresponding author on reasonable request.

## Abbreviations

IBD: Inflammatory bowel disease
CD: Crohn’ s disease
UC: Ulcerative colitis
LMR: Lymphocyte to monocyte ratio
PLR: Platelet to lymphocyte ratio
NLR: Neutrophil to lymphocyte ratio
CBC: Complete blood count
RDW: Red blood cell distribution width
ESR: C-reactive protein
CRP: Erythrocyte sedimentation rate
CDAI: Crohn disease activity index
IQR: Interquartile range
AUROC: Area under the receiver operating characteristic curve
